# Dr. Francis Delafield

**Published:** 1915-09

**Authors:** 


**Dr. Francis Delafield,** P. & S. 1863, the pathologist, a resident of N. Y. City, died of apoplexy at Noroton, Conn., July 17.

